# NLRP3 Inflammasome Modulates Post-Burn Lipolysis and Hepatic Fat Infiltration via Fatty Acid Synthase

**DOI:** 10.1038/s41598-018-33486-9

**Published:** 2018-10-12

**Authors:** Roohi Vinaik, Mile Stanojcic, Marc G. Jeschke

**Affiliations:** 1 Department of Surgery, Division of Plastic Surgery, University of Toronto, Toronto, Canada; 2 Department of Immunology, University of Toronto, Toronto, Canada; 3 Ross Tilley Burn Centre, Sunnybrook Health Sciences Centre, Toronto, Canada; 4 Sunnybrook Research Institute, Toronto, Canada

## Abstract

Burns result in generalized catabolism, lipolysis, and hyperinflammation. NLRP3 inflammasome, a mediator of hyperinflammation, is upregulated in burn patients’ adipose tissue within 7 days post-burn. However, its role during the acute phase is unknown. Here, wild-type (WT) and NLRP3 knockout (NLRP3^−/−^) mice were exposed to 25% TBSA scald burn. Flow cytometric analysis demonstrated greater liver macrophage infiltration in NLRP3^−/−^ yet decreased protein expression of NLRP3 components, ER stress, and apoptosis. NLRP3^−/−^ had increased circulating free fatty acids (FFA), fatty deposition and liver weight 1 hour post-burn. Alterations in adipose fatty acid synthase (Fasn) expression affects FFA levels post-burn; WT have an early peak in Fasn gene and protein expression that is lost in NLRP3^−/−^, resulting in increased lipolysis and hepatic fatty deposition. In summary, our findings reveal that NLRP3 inflammasome activation is a double-edged sword. While prolonged inflammation and long-term effects of macrophage activation are associated with poor outcomes, acute inflammation may be beneficial. These results highlight the important metabolic role that NLRP3 inflammasome plays in the acute phase, ultimately affecting survival post-burn.

## Introduction

Thermal injuries are responsible for an estimated 300,000 deaths per year worldwide, with approximately 11 million cases requiring medical attention^1^. Severe burns are accompanied by a host of detrimental responses, including stress-related inflammatory and metabolic changes that persist well beyond the initial trauma^2^. Although patient outcomes have improved due to implementation of established therapeutic strategies, morbidity and mortality of burn patients remain substantial and unacceptably high. Thus, a better understanding of underlying metabolic and inflammatory changes still remains.

Severe burns are accompanied by prolonged metabolic changes that include an initial “ebb phase” during which metabolic rate and tissue perfusion are decreased^3^. This response occurs during the first two to three days post-burn and is followed by the “flow phase,” which is characterized by a hypermetabolic response that persists for up to two years post-injury^3^. The flow phase involves elevated resting energy expenditure (REE), features of stress-induced diabetes, muscle catabolism and lipolysis^4^. Additionally, associated hyperinflammation, which is characterized by marked increases in pro-inflammatory mediators, eventually results in altered metabolism^4^. Hypermetabolism and hyperinflammation lead to immune dysfunction, which increases patients’ susceptibility to infection and sepsis^3^. Ultimately, these changes can lead to physiologically exhaustion and death^5^.

One of the several mediators involved in the cross-talk between post-burn inflammation and metabolic dysregulation are inflammasomes. Inflammasomes are a family of multiprotein complexes named by their nucleotide-binding and oligomerization domain-like receptor (NLR) and have an important role in innate immunity and are activated in response to stress, cell damage, tissue injury and infection^4,6^. The most studied of these, the NLRP3 inflammasome, detects pathogen-associated molecular patterns (PAMPs), inducing an inflammatory response to these danger signals^7^. This complex also detects non-infectious, damage-associated molecular patterns (DAMPs) including mitochondrial DNA, increased formation of reactive oxygen species, and circulating free fatty acids (FFA), all of which are present after severe burn^8^. NLRP3 inflammasome is also critical for metabolic regulation^4^. Recently, our group has shown that NLRP3 inflammasome is upregulated in the adipose tissue of burn patients, and its expression is positively correlated with mortality, suggesting that NLRP3 may have an imperative function during the acute phase after injury^4^.

Another consequence of metabolic dysfunction post-injury includes elevated FFA due to burn-induced lipolysis that drives fatty deposition into vital organs such as the liver^9^. Hepatic steatosis, or fatty liver, is a common post-burn complication that is implicated in poor outcomes, morbidity and mortality^9^. Interestingly, recent research in aging suggests that chronic NLRP3-driven inflammation can reduce adipocyte lipolysis^10^. Using a model of sepsis, it was recently delineated that NLRP3 inflammasome activation itself is regulated by fatty acid synthase (Fasn)-dependent lipid synthesis^11^. Fasn is a major enzyme involved in lipid biosynthesis, a process that is activated during inflammation^11^. Fasn may also have a role in post-burn inflammation, suggesting a possible link between post-burn inflammation and altered fat metabolism.

The aim of this study was to determine the role of NLRP3 inflammasome in inflammation and metabolism during the acute phase post-burn. Thus, we wanted to define whether NLRP3 inflammasome knockout would improve outcomes by dampening the initial inflammatory responses locally in tissue. Additionally, we wanted to define the role of NLRP3 inflammasome in post-burn lipolysis and hepatic fat infiltration and delineate possible mechanisms between these two burn injury complications. These results highlight the critical role of NLRP3 inflammasome in survival during the acute post-burn phase, while also shedding light on its role in altered post-burn fat metabolism and hepatic dysfunction.

## Results

### Liver weight relative to body weight is significantly elevated in NLRP3^−/−^ mice compared to WT acutely post-burn

**Figure 1:**
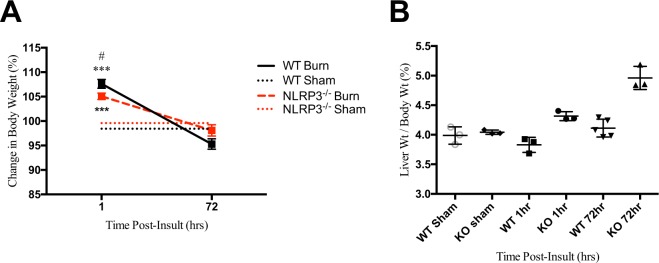
Post-burn changes in WT versus NLRP3^−/−^. (**A**) Change in body weight over time. Increased post-burn (**B**) liver weight as a percentage of body weight in NLRP3^−/−^. Values are presented as mean ± standard error. Burn versus sham ^*^p < 0.05; ^**^p < 0.01; ^***^p < 0.001, WT versus NLRP3^−/−^ burn ^#^p < 0.05; ^##^p < 0.01; ^###^p < 0.001.

When comparing body weight, both WT and NLRP3^−/−^ exhibited similar changes; weight increases relative to sham by 1 hour after injury and normalizes by the end of the acute phase (72 hours; Fig. 1A). There was no significant difference in body weight between WT and NLRP3^−/−^ by the end of the acute phase. However, we found that liver weight relative to body weight increased significantly by the end of the acute phase in NLRP3^−/−^ versus WT (4.961% vs. 4.112%, p < 0.05). Additionally, differences in liver weight were seen as early as 1 hour post-burn in NLRP3^−/−^ mice (4.32% vs. 4.04%, p < 0.01) as opposed to WT, which was relatively unchanged compared to sham (Fig. 1B). Our results suggested that lack of NLRP3 has an acute effect on the liver. Since previous work demonstrated that NLRP3 affects macrophage migration, we subsequently investigated the role of NLRP3 in liver macrophage recruitment^12^.

### Acute macrophage infiltration in liver is exclusive to NLRP3^−/−^ without corresponding increases in NLRP3 components, ER stress, or apoptosis

**Figure 2:**
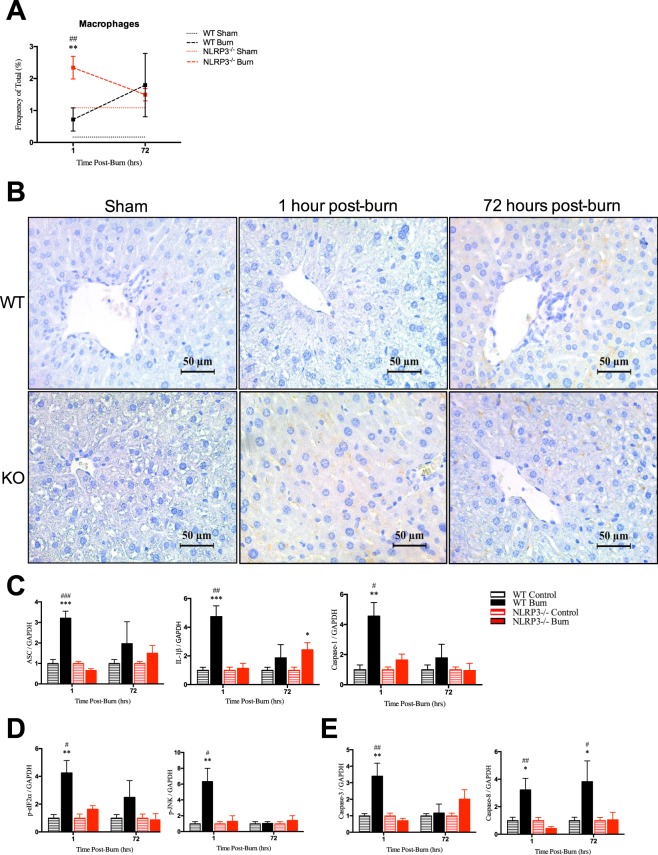
Increased macrophages acutely after burn in liver in NLRP3^−/−^ yet no corresponding increase in protein expression of NLRP3 components, ER stress, or apoptosis. (**A**) Flow cytometry for macrophage distribution and (**B**) CD11b staining for monocytes in WT and NLRP3^−/−^. Protein expression for (**C**) NLRP3 components, (**D**) ER stress and (**E**) apoptosis markers. Values are presented as mean ± standard error. Burn versus sham ^*^p < 0.05; ^**^p < 0.01; ^***^p < 0.001, WT versus NLRP3^−/−^ burn ^#^p < 0.05; ^##^p < 0.01; ^###^p < 0.001.

Using flow cytometry, we showed that NLRP3^−/−^ demonstrated a differential response acutely after (1 hour) injury compared to wild-types. During the acute phase, there is no significant post-burn liver macrophage infiltration in WT (Fig. 2A). However, at 1 hour, NLRP3^−/−^ have over a three-fold increase in macrophage infiltration compared to WT (2.34% vs. 0.72%, p < 0.001) but no significant difference at 72 hours. These results were confirmed using CD11b immunostaining in liver, which showed infiltration at 1 hour in NLRP3^−/−^ but no change between WT 1 hour post-burn and sham (Fig. 2B). Although WT initially have less macrophage infiltration relative to NLRP3^−/−^, WT demonstrate greater protein expression of NLRP3 components, ER stress, and apoptosis (Fig. 2C–E). These results suggest that macrophage recruitment to the liver may have a role in mitigating ER stress. Decreased macrophage infiltration could result in the observed ER stress responses seen in WT liver. Prolonged ER stress signaling through the unfolded protein response (UPR) eventually leads to hepatocyte cell death involving classical markers of apoptosis, including Caspase 3, which we demonstrate here as well^13^. While ER stress and apoptotic features are seen post-burn, we propose that this delay in macrophage recruitment is not the primary mechanism by which NLRP3 exerts its hepatic effects.

### NLRP3^−/−^ have increased liver fat deposition and circulating FFA

**Figure 3:**
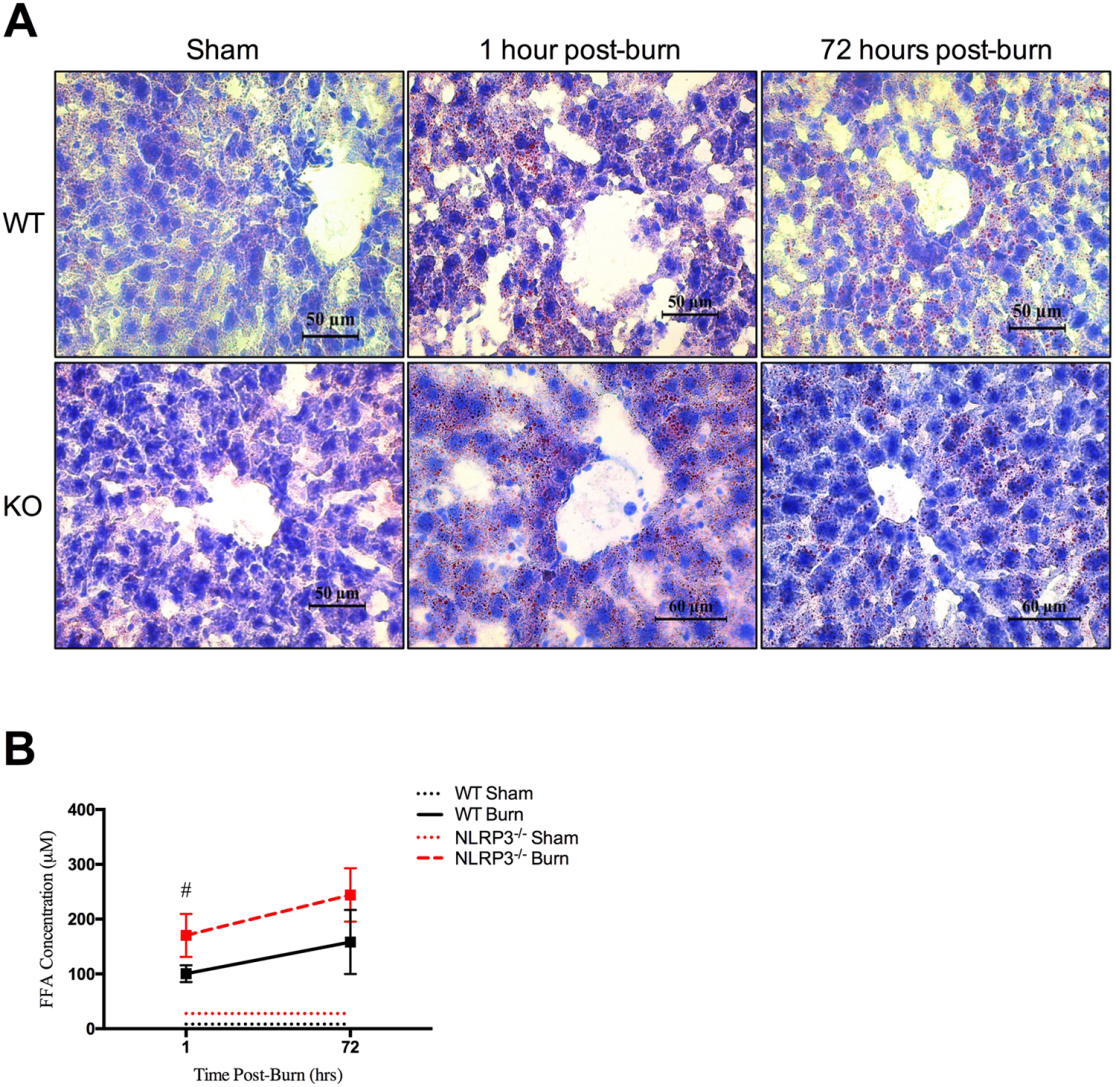
Increased circulating free fatty acids and liver fat deposition in NLRP3^−/−^. (**A**) Oil Red O staining for adipose deposition in NLRP3^−/−^ and WT mice shams, 1 hour post-burn, and 72 hours post-burn. (**B**) FFA concentration in WT and NLRP3^−/−^ 1 hour and 72 hours post-burn. Values are presented as mean ± standard error. Burn versus sham ^*^p < 0.05; ^**^p < 0.01; ^***^p < 0.001, WT versus NLRP3^−/−^ burn ^#^p < 0.05; ^##^p < 0.01; ^###^p < 0.001.

Next, we investigated the cause of post-burn differences in liver weight between WT and NLRP3^−/−^. We hypothesized that the increased liver weight seen in NLRP3^−/−^ is likely due primarily to liver fatty deposition as opposed to post-burn edema. While fatty deposition itself could increase edema by affecting capillary leakage, this is speculative. Here, we demonstrated differences in fatty deposition between WT and NLRP3^−/−^, which we further confirm with Oil Red O staining for lipid droplets (Fig. 3A). Interestingly, Oil Red O staining revealed the presence of lipids as early as 1 hour post-burn in NLRP3^−/−^, while similar extent of fatty infiltration is not seen until 72 hours in WT.

Studies in aging populations show that deletion of NLRP3 resolves the age-related decline in lipolysis seen in WT^10^. Since AT catabolism, release of FFA, and organ fatty infiltration are common post-burn, we expected that our NLRP3^−/−^ would similarly demonstrate significantly increased lipolysis compared to WT. Indeed, our results show that circulating FFAs are elevated significantly at 1 hour in NLRP3^−/−^ compared to WT (170.35 vs. 100.53 µM, p < 0.05) (Fig. 3B). Overall, our results indicate that lack of NLRP3 affects adipose metabolism as early as 1 hour post-burn, specifically by increasing circulating FFA via enhanced lipolysis. Ultimately, these fatty acids are rapidly deposited in liver, potentially disrupting liver function and resulting in hepatomegaly that can be seen by the end of the acute phase.

### Persistent increases in chemotactic cytokines in NLRP3^−/−^

**Figure 4:**
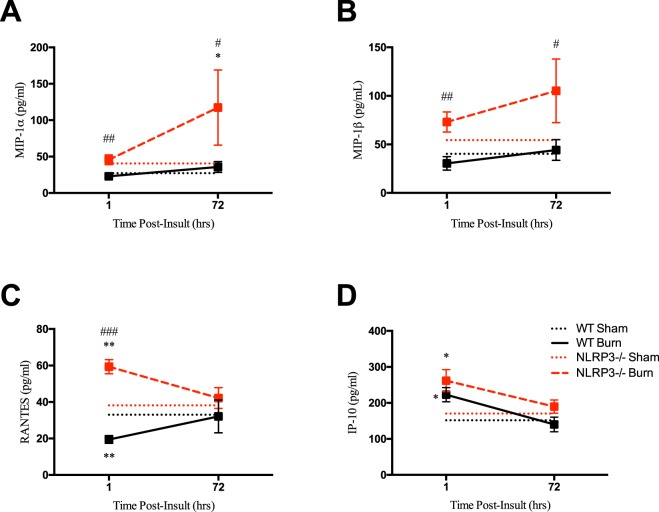
Increased acute production of chemokines in NLRP3^−/−^. Persistent increase in (**A**) MIP-1α, (**B**) MIP-1ß, (**C**) RANTES, and (**D**) IP-10 in NLRP3^−/−^. Values are presented as mean ± standard error. Burn versus sham ^*^p < 0.05; ^**^p < 0.01; ^***^p < 0.001, WT versus NLRP3^−/−^ burn ^#^p < 0.05; ^##^p < 0.01; ^###^p < 0.001.

To assess the systemic effects of NLRP3 that affect macrophage recruitment, we measured serum levels of chemokines, or inducers of chemotaxis. NLRP3^−/−^ had a greater proportion of chemokines throughout the acute phase relative to WT (Fig. 4A–D). This includes MIP-1α, MIP-1ß and RANTES, which are cytokines that are chemotactic for monocytes and have a role in inflammation. While WT show no change in MIP-1α and MIP-1ß levels over time, NLRP3^−/−^ demonstrate a progressive peak in expression at 72 hours post-burn compared to WT (MIP-1α: 117 vs. 27 pg/mL, p < 0.05; MIP-1ß 105 vs. 40 pg/mL, p < 0.05). Similar early increases in expression (1 hour) were also consistent for RANTES for NLRP3^−/−^ compared to WT (59 vs. 19 pg/mL, p < 0.001). Additionally, NLRP3^−/−^ demonstrate a similar early (1 hour) increase in type I T-helper inflammatory cytokines TNF-α and IFN-γ, which activate pro-inflammatory macrophages^12^ (Fig. S1A). There were no significant differences in traditional pro-inflammatory cytokines, such as IL-6 and IL-1ß, or anti-inflammatory cytokines (Fig. S1B,C). Taken together, our results suggest that lack of NLRP3 results in increased macrophage chemotaxis and activity after burn. Additionally, NLRP3 has a role in local inflammation rather than systemic inflammatory responses.

### Reduced macrophage recruitment and increased early expression of NLRP3 and ER stress components in AT of NLRP3^−/−^

**Figure 5:**
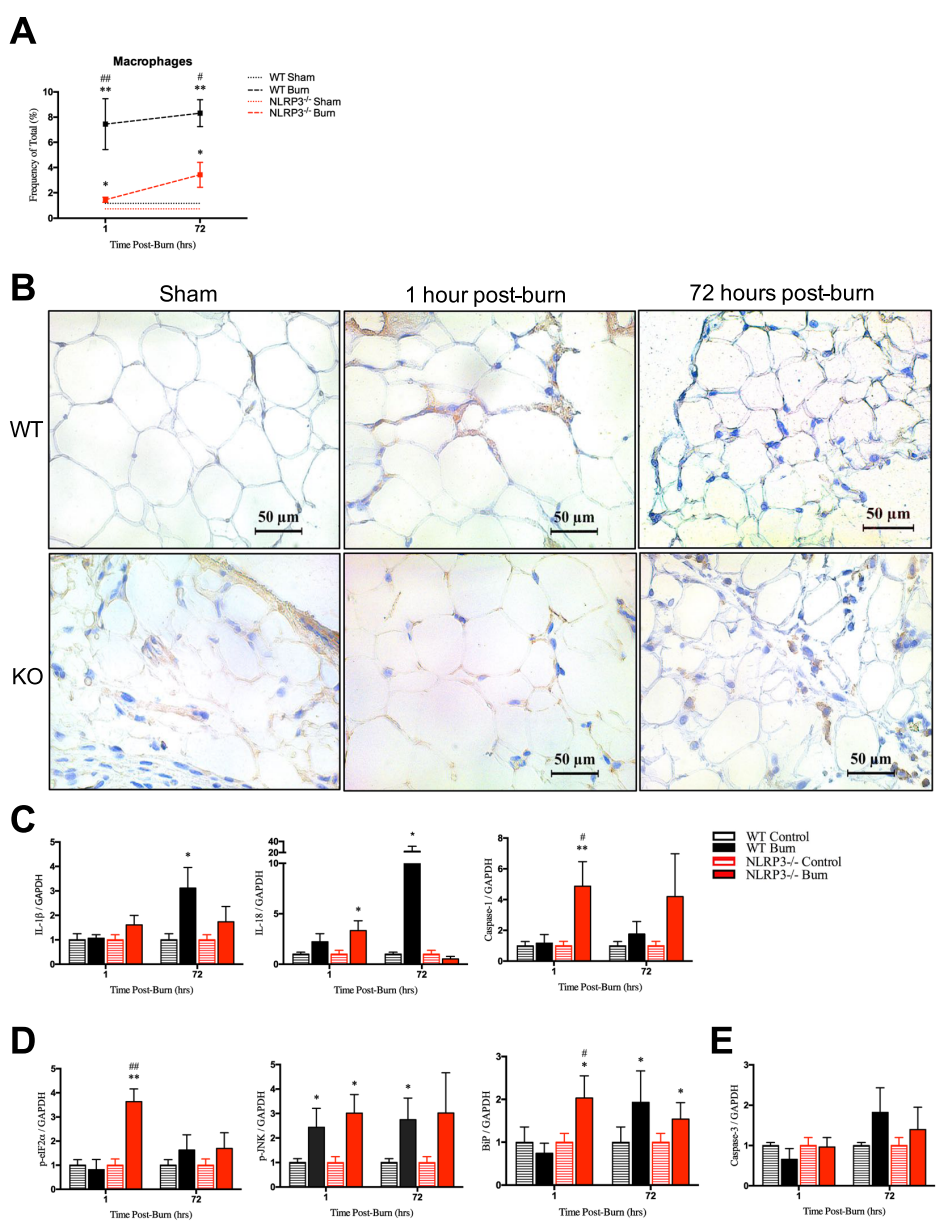
Acute increase in adipose macrophage infiltration in WT yet no consistent initial increase in WT protein expression of NLRP3 components, ER stress, or apoptosis. (**A**) Flow cytometry for adipose macrophage distribution and corresponding (**B**) F4/80 staining in WT and NLRP3^−/−^. Protein expression for (**C**) NLRP3 components, (**D**) ER stress and (**E**) apoptosis markers. Values are presented as mean ± standard error. Burn versus sham ^*^p < 0.05; ^**^p < 0.01; ^***^p < 0.001, WT versus NLRP3^−/−^ burn ^#^p < 0.05; ^##^p < 0.01; ^###^p < 0.001.

Extending the aforementioned findings, we subsequently investigated local metabolic and inflammatory changes in AT. Increased adipose lipolysis is seen in conditions of ER stress such as burns and can be attributed to direct lipolytic effects of inflammatory AT macrophages^14^. Seeing as the lack of NLRP3 resulted in acute increases in chemokines and some pro-inflammatory mediators, we wanted to determine the effect on monocyte/macrophage recruitment to AT after burn. Using flow cytometry, we showed that NLRP3^−/−^ demonstrated a differential response acutely after (1 hour) injury compared to wild-types. By 1 hour post-burn, WT showed a significant increase in macrophages compared to NLRP3^−/−^ that persists and does not change significantly throughout the acute phase (7.45% to 6.91% at 72 hours) (Fig. 5A,B). Interestingly, acute (1 hour) increases in protein expression of NLRP3 components in AT were observed in NLRP3^−/−^, not WT. Specifically, IL-1ß and IL-18 expression did increase in WT but only at 72 hours despite significant early macrophage infiltration in WT (Fig. 5C). Additionally, NLRP3^−/−^ demonstrate consistent increases in ER stress proteins 1 hour post-burn (Fig. 5D) with no significant difference in apoptosis markers over time (Fig. 5E). These results suggest that lack of NLRP3 alters macrophage migration to important immunometabolic organs such as AT after burn. Deletion of NLRP3 not only impairs macrophage migration, but in certain cases it may also reprogram macrophages towards a pro-inflammatory phenotype^12,15^. This in turn could contribute to the elevated inflammatory and ER stress responses demonstrated here, which could account for increased AT lipolysis^16^. Additionally, conditions of adrenergic activation such as burn injury induce anti-inflammatory macrophage infiltration in WT AT^17^. Taken together, this could account for that fact that WT have increased macrophage infiltration yet less inflammation, ER stress and lipolysis compared to NLRP3^−/−^ counterparts. While not statistically significant, WT may have improved survival during the acute post-burn phase, suggesting that macrophage recruitment to AT may be a necessary response after burn (Figure S2).

### NLRP3^−/−^ lack a critical peak in Fasn expression, resulting in altered fat metabolism

**Figure 6:**
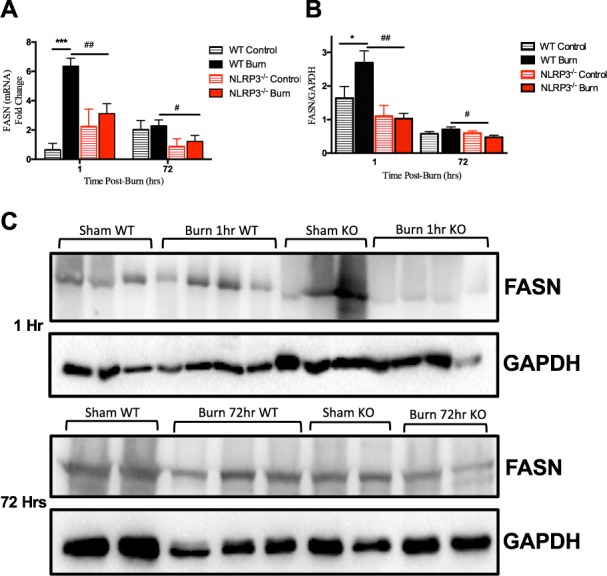
Acute increase in adipose Fasn expression is lost in NLRP3^−/−^. (**A**) Adipose Fasn gene expression and (**B**) protein expression. (**C**) Representative cropped Western blot of Fasn and GAPDH for 1 hour post-burn (top image) and 72 hour post-burn (bottom image). Values are presented as mean ± standard error. Burn versus sham ^*^p < 0.05; ^**^p < 0.01; ^***^p < 0.001, WT versus NLRP3^−/−^ burn ^#^p < 0.05; ^##^p < 0.01; ^###^p < 0.001.

Based on the previous findings, we extended our analysis to determine the underlying mediator of AT lipolysis and increased FFA in circulation in NLRP3^−/−^ relative to WT. Comparing alterations in AT gene and protein expression of Fasn, there were no significant increases in the gene expression of Fasn in NLRP3^−/−^ post-burn relative to shams. However, at 1 hour, WT had over a 9-fold significant increase in Fasn gene expression relative to sham (6.36 vs. 0.67, p < 0.001). At 72 hours, Fasn gene expression decreases but is still elevated relative to knockouts (Fig. 6A). Fasn protein expression follows a similar pattern, with significantly increased expression at 1 hour in WT, which is lost in NLRP3^−/−^ (Fig. 6B,C). While Fasn protein expression decreases by 72 hours post-burn in WT, expression is still higher in WT relative to NLRP3^−/−^. Interestingly, these findings coincide with the acute increase in FFA and liver fatty infiltration seen in NLRP3^−/−^. These results suggest that loss of adipose Fasn expression 1 hour after burn in NLRP3^−/−^ decreases lipid synthesis and instead results in liberation of FFA from adipose tissue. As a result, the circulating FFA are deposited in NLRP3^−/−^ liver, resulting in hepatomegaly by 72 hours and eventually, hepatic dysfunction.

## Discussion

NLRP3 inflammasome is the only NLRP family member that is able to recognize host danger signals (DAMPS)^18^. These signals are endogenous responses to cellular stress or damage and are seen in a variety of conditions, such gout, asbestosis, osteoarthritis, and metabolic diseases including type 2 diabetes (T2D) and obesity^18^. Previous studies on NLRP3 inflammasome in burns evolve from the fact that it is involved in T2D, which parallels post-burn stress-induced diabetes^4^. Burn patients also suffer from severe, prolonged inflammatory responses, likely initiated via NLRP3 inflammasome activation. Considering this, knocking out NLRP3 and dampening post-burn inflammatory responses putatively could have beneficial outcomes.

However, our findings suggest that mitigating early post-burn inflammation has detrimental rather than beneficial effects. We demonstrated a trend towards increased mortality in NLRP3^−/−^ compared to WT during the acute phase (≤72 hours), although not statistically significant. However, this may be due to the fact that our model was moderate burn in rodents. We previously showed that NLRP3 increases with burn severity in humans, suggesting that a more pronounced difference might be seen in a severe burn model^4^. Alternatively, although it has an important role in post-burn inflammation, lack of NLRP3 exclusively may not significantly increase mortality. Rather, it facilitates early post-burn changes that when combined with other metabolic factors, could place patients at a survival disadvantage.

Interestingly, our results suggest these changes are predominantly local rather than systemic phenomena; NLRP3^−/−^ have increased chemokine and certain pro-inflammatory cytokine levels, but no difference in systemic inflammatory markers. Notable post-burn changes include altered macrophage migration between NLRP3^−/−^ and WT to critical sites including liver and AT. In liver, increased acute (1 hour) infiltration occurs exclusively in NLRP3^−/−^ without corresponding increases in protein expression of NLRP3, ER stress, and apoptosis components; this mirrors other disease states such as NAFLD. Potentially, these infiltrating macrophages could be the anti-inflammatory sub-type, which would explain why NLRP3^−/−^ demonstrate decreased hepatic inflammation. Subsequent studies investigating the specific macrophage type would shed light on differential post-burn responses between WT and NLRP3^−/−^. However, despite decreased inflammatory and stress responses, NLRP3^−/−^ liver have increased tissue damage in the form of hepatic fatty deposition.

We showed that acute hepatic fatty deposition (1 hour) occurs in conjunction with increased circulating FFA due to enhanced lipolysis. Subsequently, we investigated local metabolic and inflammatory changes in AT, demonstrating diminished macrophage recruitment in NLRP3^−/−^. Deletion of NLRP3 can also reprogram macrophages towards a pro-inflammatory phenotype, which may contribute to the elevated inflammatory and ER stress responses seen in NLRP3^−/−^ AT^19^. These macrophages also exhibit direct lipolytic activity, which results in increased AT lipolysis seen in NLRP3^−/−^. While not a focus of this particular work, further studies confirming the specific macrophage subtype could be of utility.

Previously, NLRP3 inflammasome was shown to directly reduce lipolysis in aged mice, which is consistent with the aforementioned results. This altered fat metabolism in NLRP3^−/−^ eventually results in fatty deposition in critical organs such as the liver. One of the lipogenic enzymes involved in fat metabolism is Fasn, which we previously demonstrated is elevated in burns and inflammatory states^20^. Interestingly, recent work using a sepsis model showed that NLRP3 inflammasome activation itself is regulated by Fasn, suggesting a potential link between post-burn inflammation and altered fat metabolism^11^. We assessed AT Fasn gene and protein expression to ascertain whether NLRP3^−/−^ has an effect on the balance between AT lipolysis and lipogenesis. Alterations in adipose Fasn expression affect *de novo* lipid synthesis, shifting the balance towards lipolysis^21^. Previous work has shown that deletion of Fasn in white adipocytes enhances sympathetic nerve outflow and browning of inguinal white adipose tissue (iWAT)^21^. In burns, we demonstrated a link between increased sympathetic stimulation of adipose tissue, lipolysis and subsequent release of FFA, and browning^22^. Additionally, previous work has demonstrated catecholamine-induced lipolysis^10^. Similarly, decreased Fasn expression seen in NLRP3^−/−^ could enhance sympathetic stimulation of iWAT, which in turn may increase lipolysis and levels of FFA. Liberated FFAs induce inflammatory responses and alter insulin sensitivity, potentially resulting in metabolic disorders and increased risk of sepsis^8^. Here, we demonstrate an acute (1 hour) peak in Fasn gene and protein expression in WT, which is lost in NLRP3^−/−^. Interestingly, these results coincide with the acute increase in FFA and liver fatty infiltration seen in NLRP3^−/−^. Our data suggests that loss of adipose Fasn expression 1 hour after burn in NLRP3^−/−^ results in predominant lipolysis rather than lipogenesis, releasing FFAs into circulation. This not only increases the risk of metabolic disease and sepsis, but also results in liver fat deposition. Ultimately, hepatic fat accumulation causes organ dysfunction, further predisposing to poor outcomes in NLRP3^−/−^. These results demonstrate one of the multiple factors that contribute to increased mortality seen in NLRP3^−/−^ during the acute phase.

In this study, we established a link between post-burn acute inflammation and metabolic alterations. This is the first study connecting NLRP3 inflammasome to the key lipogenic regulator, Fasn, in burns. While previous work demonstrated that Fasn regulates NLRP3 activation, we show here that NLRP3 in turn regulates Fasn expression, suggesting a possible feedback mechanism^11^. Unexpectedly, these changes occur as early as 1 hour after burn and eventually have downstream metabolic effects at the organ level. The relationship between NLRP3 and Fasn has implications in inflammation, infection, and metabolism, highlighting the importance of studying these two factors.

A limitation of this work is that this is a rodent study with only male mice. However, previous work demonstrated that females and males have similar post-burn outcomes^23,24^. Additionally, while this is a rodent study, it has broader clinical applications as well. In the clinical setting, the NLRP3 inhibitor glibenclamide is given as a blood glucose regulator in burn patients^25^. Extrapolating these results to humans, our data suggests that glibenclamide should not be given initially after thermal injuries as it would mitigate inflammation during the acute phase. However, chronic inflammation is detrimental in disease states such as obesity and similarly, prolonged post-insult inflammatory responses are likely a cause of poor outcomes in burns. Since the data presented here pertains to acute NLRP3 activation, further studies focusing on longitudinal NLRP3 expression are required because metabolic events primarily present days to weeks later. Potentially, delayed inhibition of inflammation and burn outcomes could be of interest in this patient population.

## Methods

### Animals and model

Animal experiments were conducted in accordance and approved by the Sunnybrook Research Institute Animal Care Committee (Toronto, Ontario, Canada). Wild-type C57/B6 (WT) and NLRP3 knockout (NLRP3^−/−^) male mice (6–8 weeks old, n = 5 per group) were purchased from Jackson Laboratories (Bar Harbor, ME) and housed at ambient temperature and cared in accordance with the Guide for the Care and Use of Laboratory Animals. All mice were anesthetized with 2.5% isoflurane and shaved along the dorsal spine region. Ringers lactate (2–3 mL) was injected subcutaneously in all treatment mice to protect the spine and buprenorphine (0.05–0.1 mg/kg body weight) was injected for pain management. A full-thickness, third degree dorsal scald burn encompassing 20–25% total body surface area (TBSA) was induced by immersing mice in 98 °C water for 10 seconds. Mice were sacrificed at the beginning (1 hour post-burn) and end (72 hours post-burn) of the acute phase. Liver, adipose, and skin (site of injury) were harvested for further analysis and blood collected following cardiac puncture. Sham mice (control) underwent identical experimental procedures, with the exception of the burn injury. All tissues were harvested upon sacrifice and stored in −80 °C until analysis.

### Cytokine profile

Rodent EDTA-anticoagulated blood samples were collected from all mice at the time of sacrifice and stored in −80°C until analysis. Blood samples were centrifuged for 20 minutes at 3,000 rpm at 4 °C. Plasma samples were used to compare inflammatory, chemokine and immune mediators between groups using a Multiplex platform (Millipore, MA). Experimental kits were all conducted in accordance with manufacturers’ protocol. Raw data was processed using Millipore Analyst software. All values are presented as mean ± SEM and expressed in pg/ml.

### Flow cytometry

Site of injury, adipose tissue, and liver were minced and digested with collagenase (Life Technologies) for 45 minutes at 37 °C while shaking. The digested cell suspension was filtered and centrifuged at 1800 rpm for 5 minutes to separate the stromal-vascular fraction. Pelleted cells were resuspended in FACS buffer (PBS containing 5% FBS and 1% L-glutamine) and passed through a 40 µM strainer (BD Bioscience) to remove large cellular debris. Cells were stained with monoclonal antibodies on ice for 30 minutes. Samples were than washed and analyzed using BD LSR II Special Order System (BD Biosciences, San Jose, CA, USA). Cells were gated on FSC-A and SSC-A, followed by doublet exclusion (FSC-W x FSC-H, SSC-W x SSC-H). The total percentage of monocytes/macrophages was identified using the following flurochrome- conjugated antibodies: anti-CD45 (anti-mouse PE-Cyanine7, eBioscience), anti-CD11b (anti- mouse Alexa APC-eFluor® 780, eBioscience), and anti-F4/80 (anti-mouse FITC, eBioscience) in accordance with the manufacturer’s flow cytometry protocol. The gating strategy for assessing innate immune cell distributions included leukocytes initially gated based on granularity and CD45 (side scatter x CD45), followed by size (forward scatter). Gated cells were stained for cell surface markers for monocytes and macrophages (CD11b^+^/F4/80^+^). Monocytes/macrophages were also gated on CD45^+^/CD11b^+^/F4/80^+^/Ly6C^+^ populations and showed similar cell proportions and trajectories so all subsequent analysis was continued using CD45^+^/CD11b^+^/F4/80^+^ gating for this population.

### Histology and immunohistochemistry

Skin, inguinal white adipose, and liver tissue collected were immediately fixed in 10% formalin and then maintained in 70% ethanol before paraffin embedding. Subsequently, tissues were sectioned and incubated with F4/80 (Abcam, #100790) for skin and adipose macrophages and CD11b (BioLegend, #101202) for liver monocytes followed by DAB staining. For Oil Red O, tissues were coated with OCT (optimal cutting temperature compound), placed on dry ice and stored at −80 °C until further analysis. Frozen tissue blocks were sectioned 10 μm thick, mounted on slides and fixed in formaldehyde (40%) for 1 min. The slides were stained with Oil Red O for 10 min. at room temperature, rinsed with water and stained using Gill’s haematoxylin for 1 min. Imaging was performed on a LSM confocal microscope (Zeiss, Germany).

### Free Fatty Acid (FFA) in Sera

FFA in the blood was determined using FFA colorimetric assay kits according the manufacturer’s instructions (Cayman Chemical).

### Gene expression Using RT-PCR

Total RNA isolated from adipose tissue was analyzed by quantitative real-time polymerase chain reaction (RT-PCR). RNA was isolated from tissue and cells using TRIzol-chloroform (Life Technologies) with subsequent purification using the RNeasy Kit (Qiagen) according to the manufacturer’s instructions. RNA (2 mg) was transcribed to cDNA using the high-capacity cDNA reverse transcription kit (Applied Biosystems). RT-PCR was performed using RNA was extracted from rodent adipose tissue using Trizol (Invitrogen, CA, USA). Reverse transcription were performed with high-capacity cDNA reverse transcription kit (ABI, MA, USA). RT-PCR was performed using the Applied Biosystems Step One Plus Real-Time PCR System. Primer sequences used are available upon request. Gene expression was expressed relative to β-actin. Due to inter-species variability between WT and NLRP3^−/−^ control concentrations, all values are presented as a ratio relative to the mean concentration of species- specific controls for a given primer.

### Western blotting

Proteins from rodent adipose tissue was extracted in RIPA buffer containing phosphatases and proteases inhibitor cocktails (Roche). Protein concentrations were determined by the BCA protein assay kit (Pierce, Mississauga, ON, Canada). Proteins were resolved by SDS-PAGE followed by western blotting using the following antibodies at 1:1000–1:5000 concentration: Fasn (Cell Signaling, MA) and GAPDH (Cell Signaling, MA, USA). Species appropriate secondary antibodies conjugated to horseradish peroxidase (BioRad, Mississauga, ON, Canada) were used and proteins visualized by enhanced chemiluminescence using the BioRad ChemiDoc MP Imaging System. Band intensities were detected, normalized and quantified with the ChemiDoc and Image Lab 5.0 software (BioRad Laboratories, Hercules, CA). Antibody concentrations are expressed relative to GAPDH. Due to inter-species variability between WT and NLRP3^−/−^ control concentrations, all values are presented as a ratio relative to the mean concentration of species-specific controls for a given antibody.

### Statistical analysis

All data are represented as mean ± SEM. Survival curves were analyzed using the log-rank (Mantel–Cox) test. Statistical analysis was performed using student’s t-test, one and two-way ANOVA and Mann–Whitney U test to compare groups, where appropriate. All graphs were created using Graphpad Prism 6.0 (San Diego, CA) and analyzed statistically using SPSS 20 (IBM Corp., NY, NY), with significance accepted at p < 0.05 (*), p < 0.01 (**), p < 0.001 (***) and p < 0.05 (#), p < 0.01 (##), p < 0.001 (###), where appropriate. Asterisks refer to a comparison between burn groups and corresponding shams, and hashtags refer to a comparison between NLRP3^−/−^ and WT.

## Electronic supplementary material

Supplemental Figures

## Data Availability

All data generated or analyzed during this study are included in this published article (and its Supplementary Information files).
