# Supplementary material for: NLRP3 Inflammasome Modulates Post-Burn Lipolysis and Hepatic Fat Infiltration via Fatty Acid Synthase
Source: Sci Rep. 2018 Oct 12;8:15197. doi: 10.1038/s41598-018-33486-9 (PMC6185951; doi:10.1038/s41598-018-33486-9)
Supplement: Supplementary file 1 — Supplemental Figures [file 41598_2018_33486_MOESM1_ESM.pdf]

NLRP3 Inflammasome Modulates Post-Burn Lipolysis and Hepatic Fat Infiltration via Fatty Acid Synthase

Roohi Vinaik MD[#4], Mile Stanojcic PhD[#4] and Marc G. Jeschke MD PhD*[1,2,3,4]

[1]Department of Surgery, Division of Plastic Surgery, University of Toronto, Canada

[2]Department of Immunology, University of Toronto, Canada

[3]Ross Tilley Burn Centre, Sunnybrook Health Sciences Centre, Toronto, Canada

[4]Sunnybrook Research Institute, Toronto, Canada

*Corresponding Author: Marc G. Jeschke, MD, PhD, Director Ross Tilley Burn Centre, Sunnybrook Health Sciences Centre; Division of Plastic Surgery, Department of Surgery, Department of Immunology, University of Toronto; Sunnybrook Research Institute, 2075 Bayview Ave., Rm. D704, Toronto, ON, CANADA, M4N 3M5

Tel: 416-480-6703; Fax: 416-480-6763; e-mail: marc.jeschke@sunnybrook.ca

Address for Reprints: Same as corresponding author, please see above

Conflicts of Interest and Source of Funding: This study was supported by - Canadian Institutes of Health Research # 123336. CFI Leader's Opportunity Fund: Project # 25407 NIH RO1 GM087285-01

[#]*These authors contributed equally*

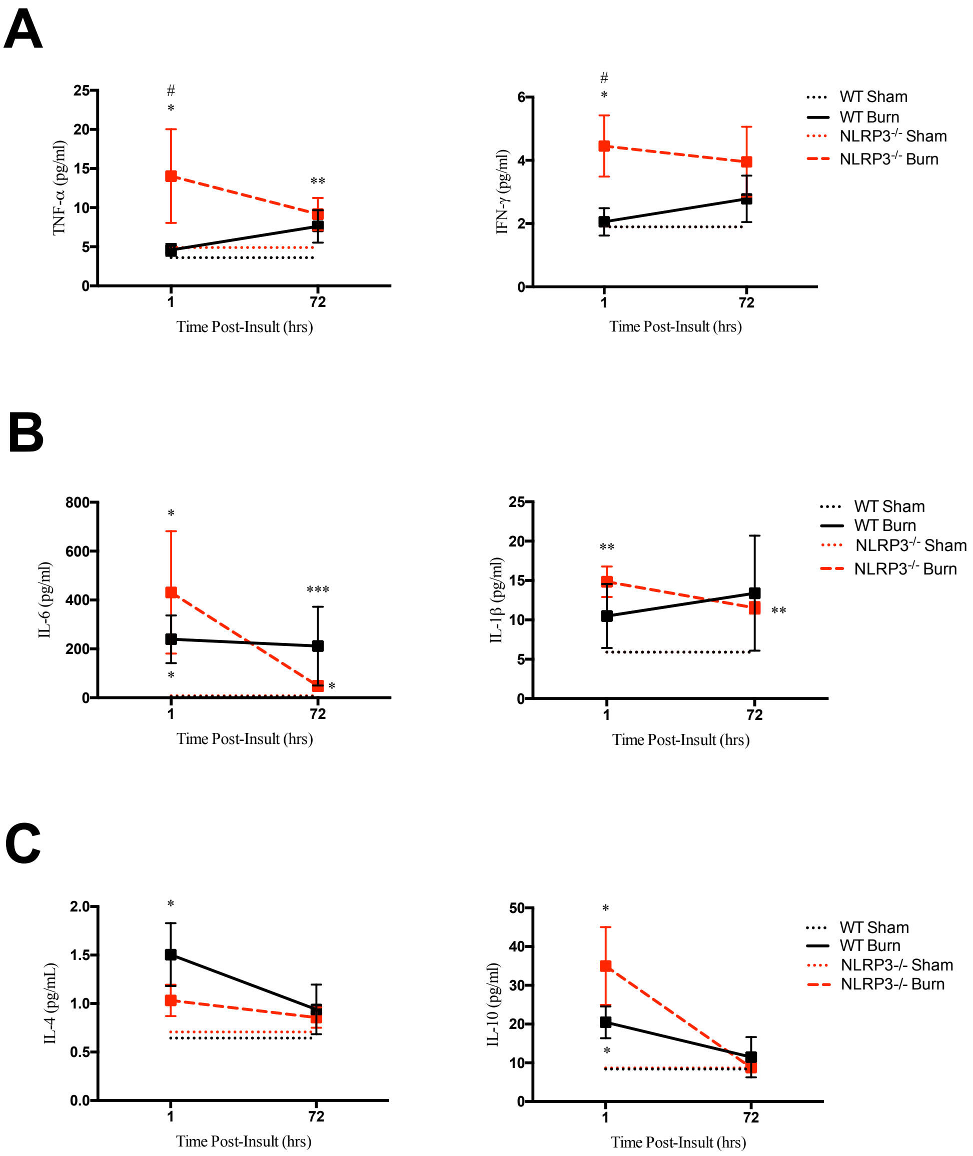

Supplemental Figure 1 Increased production of certain cytokines in NLRP3$^{-/-}$. (**A**) While certain inflammatory cytokines are systemically increased in NLRP3$^{-/-}$, there are no differences in (**B**) classic cytokines. A similar pattern was seen in (**C**) anti-inflammatory cytokines. Values are presented as mean ± standard error. Burn versus sham $^{*}p < 0.05$; $^{**}p < 0.01$; $^{***}p< 0.001$, WT versus NLRP3$^{-/-}$ burn $^{\#}p < 0.05$; $^{\#\#}p < 0.01$; $^{\#\#\#}p < 0.001$.

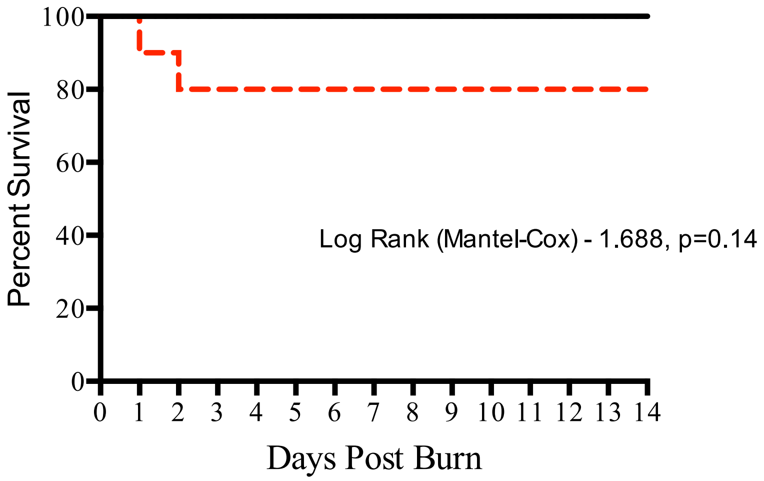

Supplemental Figure 2 Survival Curve for WT versus NLRP3$^{-/-}$.

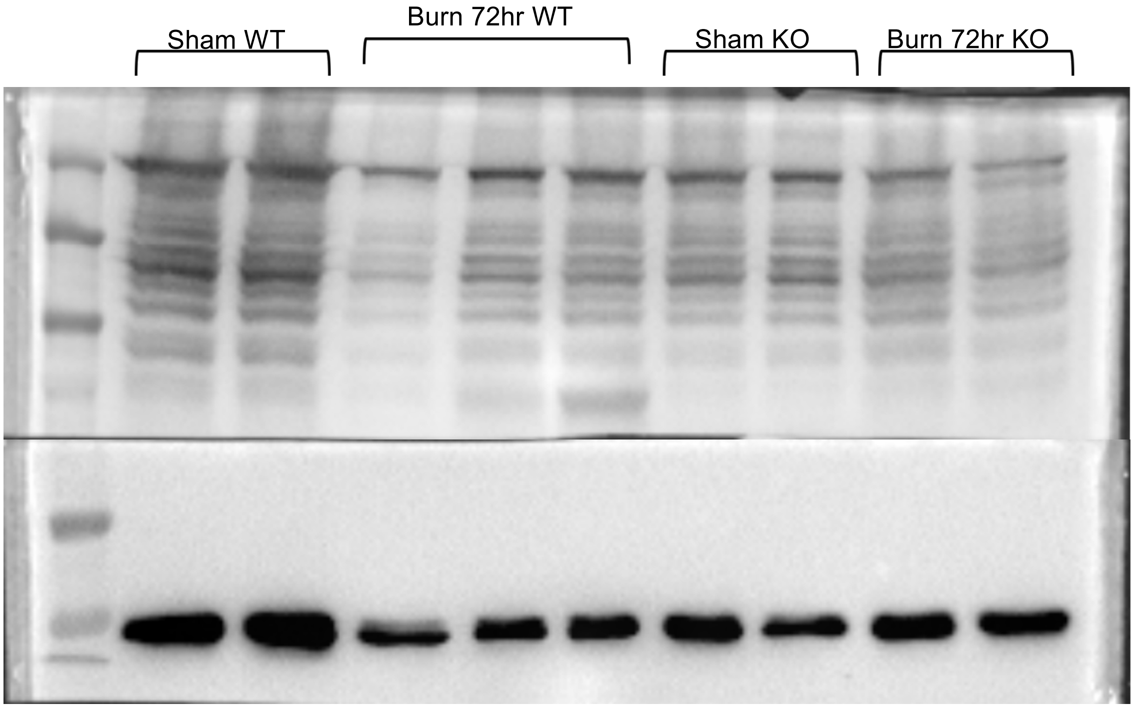

Representative Western blot image for adipose Fasn (top panel) and GAPDH (bottom panel) at 72 hour post-burn in burn groups. Membrane was cut below 75kDa, but images were taken from the same membrane.

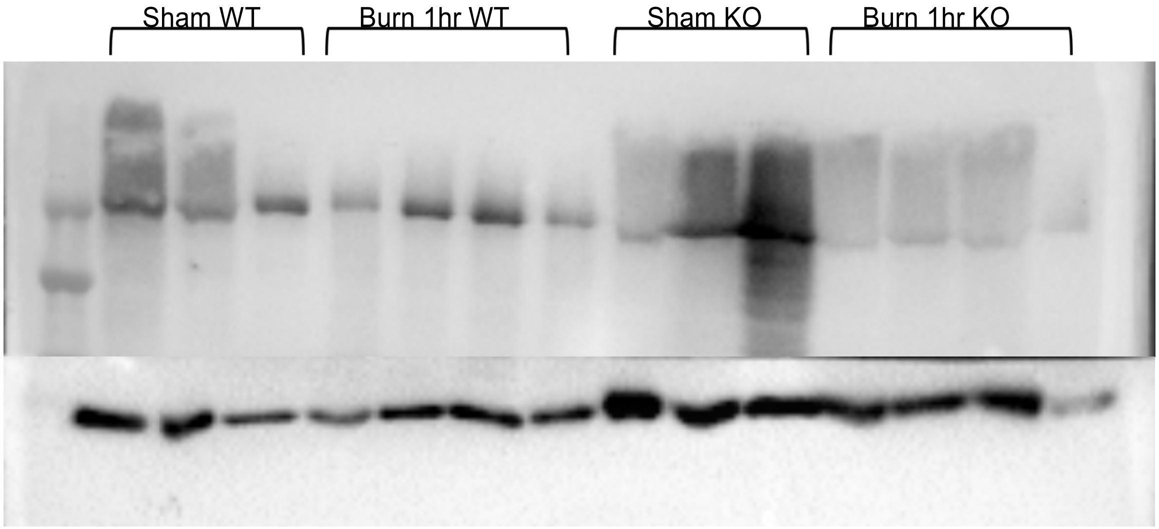

Representative Western blot image for adipose Fasn (top panel) and GAPDH (bottom panel) at 1 hour post-burn in burn groups. Membrane was cut at 100 kDa, but images were taken from the same membrane.
